# Using Recurrent Neural Networks to Reconstruct Temperatures from Simulated Fluorescent Data for use in Bio-Microfluidics

**DOI:** 10.21203/rs.3.rs-3311466/v1

**Published:** 2023-09-04

**Authors:** Jacob Kullberg, Derek Sanchez, Brendan Mitchell, Troy Munro, Parris Egbert

**Affiliations:** 1 Computer Science Department, Brigham Young University, 3361 TMCB, Provo, 84602, UT, USA.; 2 Mechanical Engineering department, Brigham Young University, 3361 TMCB, Provo, 84602, UT, USA.

**Keywords:** Fluorescence, LSTM, Microfluidic devices, Temperature Reconstruction

## Abstract

Many biological systems have a narrow temperature range of operation, meaning high accuracy and spatial distribution level are needed to study these systems. Most temperature sensors cannot meet both the accuracy and spatial distribution required in the microfluidic systems that are often used to study these systems in isolation. This paper introduces a neural network called the Multi-Directional Fluorescent Temperature Long Short-Term Memory Network (MFTLSTM) that can accurately calculate the temperature at every pixel in a fluorescent image to improve upon the standard fitting practice and other machine learning methods use to relate fluorescent data to temperature. This network takes advantage of the nature of heat diffusion in the image to achieve an accuracy of ±0.0199 K RMSE within the temperature range of 298K to 308 K with simulated data. When applied to experimental data from a 3D printed microfluidic device with a temperature range of 290 K to 380 K, it achieved an accuracy of ±0.0684 K RMSE. These results have the potential to allow high temperature resolution in biological systems than is available in many microfluidic devices.

## 1 Introduction

Many biological systems need high levels of accuracy to study that current temperature sensors cannot meet [1]. For instance, microfluidic devices are becoming an increasingly important technology in the field of biological analysis and precision medicine [2]. The micro size of the microfluidic devices causes significant problems in observing and measuring thermal interactions on the chip because it can be challenging to use traditional temperature sensing probes. Because of this, most methods used to measure the spatial distribution of temperature within microfluidic devices use optical sensing [3], with fluorescent thermometry being one of the most straight-forward approaches to implement [4]. This technique can also be used to image temperature throughout the entire microfluidic device [5, 6], as the organic fluorescent dye [7], rare earth and transition metal ions [8], and nanocrystals [9] (such as quantum dots [10, 11]) can easily be incorporated into the microfluidic device. One critical shortcoming is that traditional analysis techniques (curve fitting to intensity or photoluminescent lifetime) to relate the fluorescent signal to temperature often limit the accuracy to near ±1 K [3, 8, 10, 12, 13]. If the temperature accuracy could be reduced by an order of magnitude (±0.1 K), the technique could be applied more widely.

Machine learning via neural networks (NNs) has the potential to determine the non-linear relationships between fluorescent spectra and the temperature of the fluorescent particle. This hypothesis is based on planar temperature reconstruction from a series of images taken using thermochromic liquid crystals (TLC), where a pixel-to-pixel based simply connected feedforward network (P2P-SCFFNN) achieved a mean absolute deviation near ±0.1 K over a 4.4 K range from 291.1 to 295.5 K [14]. NNs have been used to recreate temperature from other signals as well, such as IR thermometry of a point [15], atmospheric temperature at varying altitudes [16], flame temperature and composition [17–19], and time-of-flight for ultrasonic waves [20]. Previous works’ fundamental limitations are that they cannot create a 2D image or have not been applied to fluorescence thermometry.

**Fig. 1:**
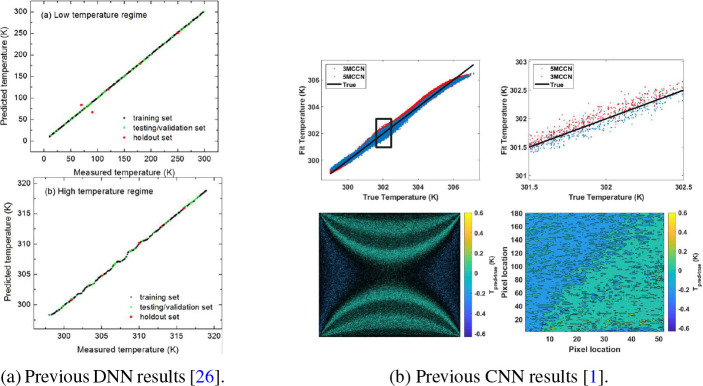
Results of previous work on using a fluorescent signal to determine temperature.

There have been limited studies on temperature reconstruction from fluorescent data. There are several papers that are zero-dimensional studies, namely temperature reconstruction at a single point. The few papers that present temperature reconstruction from fluorescent data generally use simply connected feed-forward networks (SCFF) trained on features of the emitted fluorescent spectra to recreate the temperature at a single illumination location point [21–24]. One network achieved an RMSE of ±0.3 K or 35 mK·Hz^−1/2^ by using the intensities of multiple spectral bands, which was twice as accurate as work Sarmanova [25] performed that used the NN to determine both temperature and pH. By training a deep neural network (DNN) on both the spectral intensity of fluorescence and the lifetime of the excited molecule [26], we have previously been able to obtain an accuracy of ±0.4 K over a 100–300 K range (Figure 1a). The key limitation of this approach is that it took several minutes to measure a single point because of the thousands of laser pulses needed to create an entire lifetime curve. This would take far too long to raster across a sample to create an image. The most current work [1] used convolutional neural networks (CNN), where we used a variant of a CNN to obtain an RMSE of ±0.15 K while using simulated fluorescent data from a microfluidic device [24] (Figure 1b). If the simulated results could be applied to an experimental system, this level of accuracy would meet the requirements to determine slight differences in the melting temperature of DNA based on the presence of mutations [27, 28]. Additionally, the accuracy could be further improved by developing neural networks whose outputs are constrained based on physical laws such as heat diffusion.

The reconstruction of temperature fields or velocity fields using neural networks that are able to be constrained by physical laws is a developing field [29]. By leveraging known physical relationships (such as the heat diffusion equation), Physics Informed Neural Networks (PINNS) attempt to recreate temperature distributions from a limited number of temperature probes [30–32] or inversely, predict where to place a limited number of temperature probes for improved measurements [29]. However, these have not been applied to the problem of using a NN to improve accuracy by including physical laws within the network to overcome issues with noise in the temperature measurement. Because heat diffuses in a regular manner, knowing a pixel’s temperature can help us calculate the temperature of the adjacent pixels. To take advantage of this, a Long Short-Term Memory (LSTM) architecture is used. An LSTM network is a type of recurrent network and is one of the most cited architectures [33]. Using the network’s short-term and long-term memory, the LSTM can track critical information along the sequence while avoiding the gradient explosion that some recurrent neural networks experience.

This paper presents a series of NNs that seek to emulate physics-informed NNs (PINNs, which bound networks outputs based on physical laws [34]) by developing a new architecture for LSTM networks [35]. Specifically, three different Fluorescent Temperature LSTM models (FTLSTM) are presented. The first type tested is an LSTM model with multiple LSTM cells. The second model is a Bi-Directional LSTM (BFTLSTM). The third model explored is the Multi-Directional LSTM (MFTLSTM). The model is designed for multi-dimensional information such as images. This emulation is possible in temperature reconstruction because the heat diffusion equation requires that temperature pixels are spatially related, and the LSTM can capture this behavior without requiring the form of the partial differential equation that PINNs do. Finally, these networks will be applied to experimental data collected from a microfluidic, fluorescent thermometry set up to demonstrate the applicability of NNs to improve temperature reconstruction.

## 2 Methods

This section will discuss the development of LSTM models with their associate Dataset #1 and the experimental setup and COMSOL multiphysics modeling used to produce Dataset #2.

### 2.1 Development of LSTM Models from Literature, Dataset #1

#### 2.1.1 Development of Dataset #1

To determine the efficacy of LSTM models when applied to reconstructing temperature from fluorescent images, we followed the previous process [1] that was used to study convolutional (CNN) and U-net networks. This would provide a baseline to determine the validity of the LSTM approach. This process resulted in a series of synthetic fluorescent images created from known temperature distributions (termed RvsZ and XvsY maps), which were analytical solutions to the heat diffusion equation. We will refer to this synthetic dataset as Dataset #1. During the creation of the temperature images, Gaussian white noise with a standard deviation of ±0.1 K was added to each pixel. The fluorescent spectra of CdSe/ZnS quantum dots (QDs) as a function of temperature was extracted from Ref [24] and summed over five spectral bands. The minimum and maximum fluorescent intensities of the five bands were scaled to grayscale values of 0 and 65,535, respectively, followed by relating the temperature of the spectra collected to grayscale fluorescent intensities. After fitting a 2^nd^ order polynomial to the data, grayscale-temperature relationships were used to generate multi-channel images (similar to a camera’s RBG channels) from the known temperature distributions. These multi-channel images mimic the behavior of our experimental system that imaged the fluorescence emitted from QDs within a microfluidic device with a camera and a filter wheel, which is described in Section 2.2. However, they are not a 1-to-1 comparison as the summation of these fluorescent bands for Dataset #1 had sharp cut-offs of the intensity at the edges of the selected wavelengths of the filter, while the images using the different fluorescent filters for Dataset #2 had more gradual transitions that resulted in images with different filters containing overlapping regions of measured wavelengths.

**Fig. 2:**
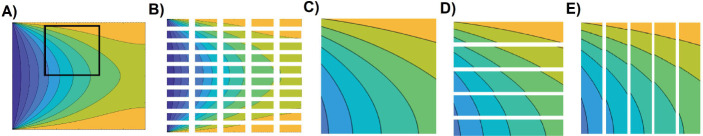
Splitting an image into sequences. A) The original image. B) Splitting into 1×5 rows. C) A sub-image from A. D) Splitting sub-image into rows. E) Splitting A sub-image into columns.

From these fluorescent images, training data for the LSTMs were created. There were two different styles of training data created. The first was linear sequences of pixels. The fluorescent images were split into 1×5 rows of pixels as shown in Figure 2. A sequence of 5 pixels was chosen for several reasons. A smaller sequence size could create significantly more unique training data from the fluorescent images than from a more extensive sequence. This would help prevent over-training of the network. The second was to keep the input size consistent throughout each network tested. As the networks start scanning in more directions, the amount of time and memory needed quickly grows. A five-pixel sequence was considered significant enough to be helpful in the smaller networks but not too large as to cause memory or time issues with the more complex LSTMs.

#### 2.1.2 Description of Networks

**Fig. 3:**
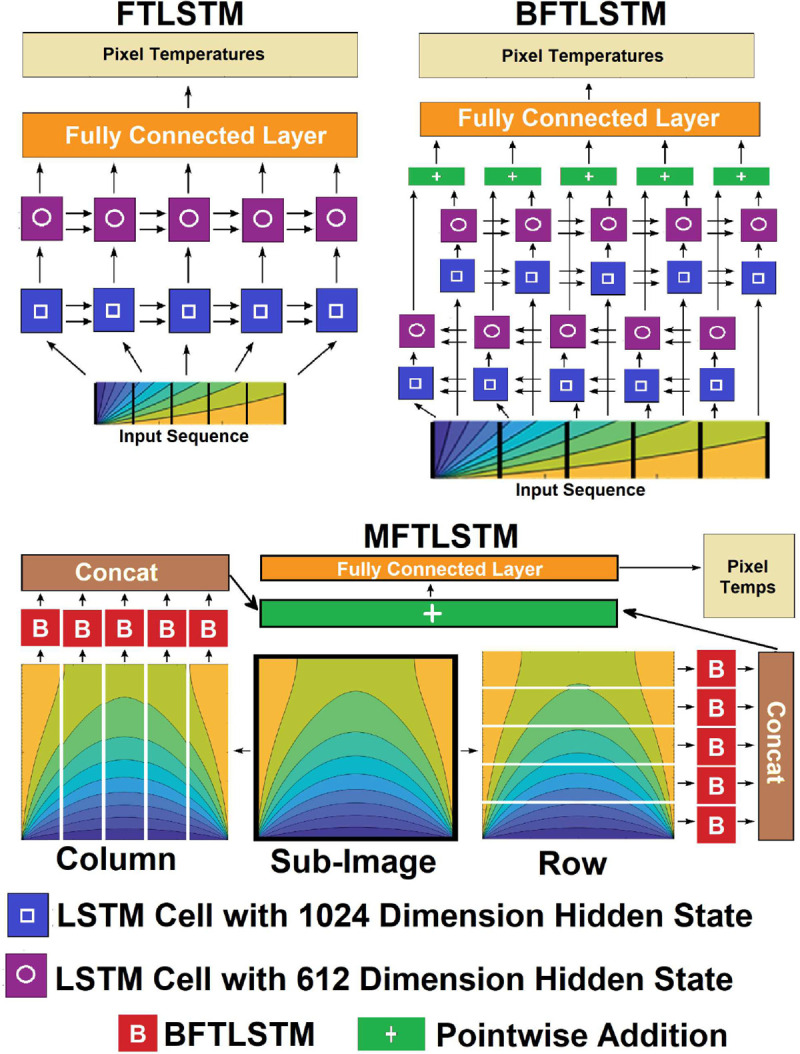
Structure of the three types of Fluorescent Temperature Long Short Term Memory Neural Networks

The first of the three networks created was the basic FTLSTM (Figure 3, top left). This network only scanned the fluorescent data in one direction. The FTLSTM took the linear sequences of pixels and their associated fluorescent data as input. The sequence was fed into an LSTM layer with a hidden state dimension of 1024. The results from this LSTM layer were then fed into a second LSTM layer with a hidden state dimension of 612. Finally, the results of this second layer were fed into a fully connected layer with a 0.2 dropout layer. The output was a sequence of temperatures corresponding with the input sequence of pixels.

The second network tested was an improved version of the FTLSTM called the Bi-Directional FTLSTM or BFTLSTM (Figure 3, top right). It is similar to the FTLSTM, except it scanned the input sequence in both the forward and reverse direction instead of just the forward direction. This is accomplished by feeding the forward and reverse sequences into separate FTLSTMs that do not have the final fully connected layer. The results from these two FTLSTMs are then pointwise added together. The resulting information is then fed into a fully connected layer with a 0.2 dropout layer. The output is also a sequence of temperatures corresponding to the input sequence of pixels.

The last network tested was built up on BFTLSTM. This was the Multi-Directional FTLSTM or MFTLSTM (Figure 3, bottom). The MFTLSTM has an input of a 5×5 sub-image of the fluorescent image (Figure 2 C). This 5×5 image is split into five columns and five rows (Figure 2 D–E). Each row and column is fed into a BFTLSTM that is missing the final fully connected layer. The column results are concatenated together. The row results are also concatenated together. The row and column results are then added pointwise to each other. The resulting vector is fed into a fully connected layer with a 0.2 dropout layer. The output of the MFTLSTM is a 5×5 matrix with temperatures corresponding to each pixel in the input 5×5 fluorescent sub-image.

### 2.2 Experimental System, Data, and Associated Images

**Fig. 4:**
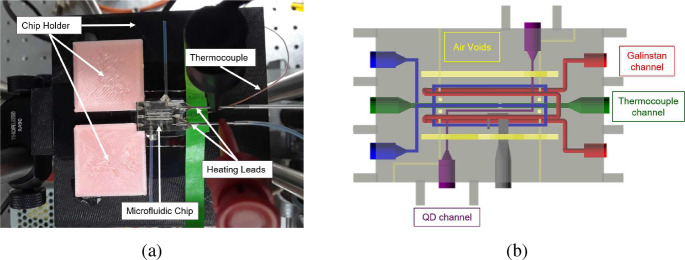
a) Image of experimental setup with microfluidic chip (device), support structures to fix its location, heating leads that were connected to galistan metal that had been inserted into the chip, and the thermocouple used to measure temperature. b) CAD model of the 3D printed microfluidic chip, highlighting key features of the device. QD channel contains PEGDA resin impregnated with quantum dots, the galinstan channel provides heating, and the thermocouple channel has a pinched portion to ensure the thermocouple location is precisely known and is in good contact with the chip.

**Fig. 5:**
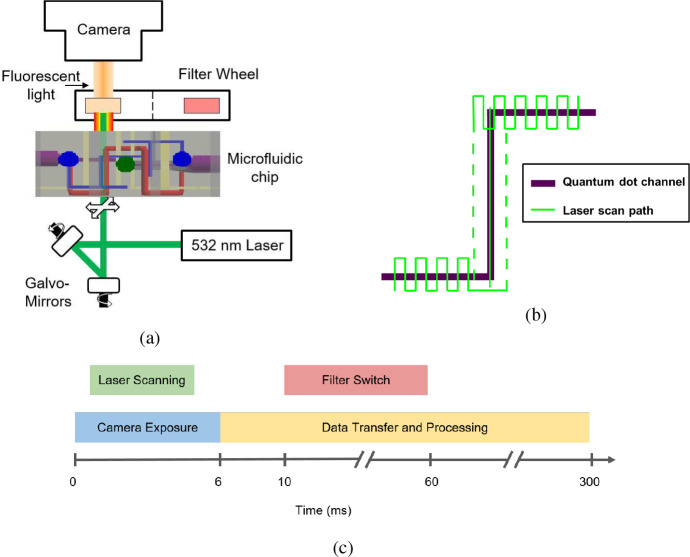
a) Schematic of the optical system used to induce and measure fluorescence from QDs. b) Schematic of the path the laser traveled to illuminate the QD channel, see Figure 4. c) Timing sequence used to acquire the fluorescent image of the microfluidic chip.

**Fig. 6:**
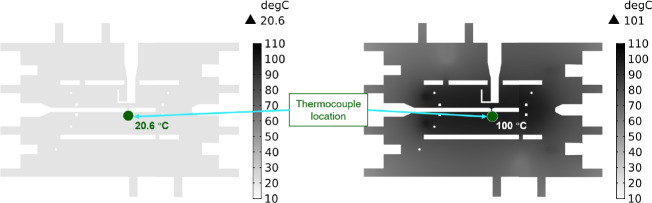
Results of COMSOL model showing the temperature distribution within the chip when the thermocouple is at 20.6 °C and 100.0 °C

As a practical demonstration of the LSTM models developed, experimental measurements of temperature-dependent fluorescence of QDs within a 3D printed microfluidic device were taken (Figure 4). Because the QD-impregnated resin was embedded within the device, an IR image of the surface of the device could not be used to determine the spatial distribution of temperature to establish a valid ground truth to train the LSTM. To overcome this limitation, we followed the process in Ref [36] where the knowledge of temperature at a single point is combined with precise thermal modeling to represent the spatial distribution around that point. Using previous validated COMSOL multiphysics models [37], we modeled the thermal environment of the experimental setup shown in Figures 4a and 5 and determined the temperature distribution within the 3D printed device (Figure 4b). The model was adjusted until the predicted temperature matched the measured temperature of the thermocouple that was located within the device (green dot in Figure 6). The following subsections describe that process. This dataset is termed Dataset #2.

#### 2.2.1 Experimental Setup

Digital Light Processing Stereolithography (DLP-SL) was used to produce a microfluidic device, also called a chip. A custom 3D printer (with an XY resolution of 7.6*μ*m and a layer size of 10*μ*m [38]) with a custom polyethylene glycol diacrylate (PEGDA) resin formulation (98 %wt PEGDA, 1 %wt Irgacure, 1 %wt Avobenzone [39]) allowed a chip with microscale voids/channels to be created for the insertion of QDs, liquid metal heaters, and a thermocouple (Figure 4a). Air voids were included within the printed chip to provide a more uniform temperature throughout the device (yellow in Figure 4b).

After printing, the channels were cleared with isopropyl alcohol and a vacuum pump. Stainless steel tubes are added to the heater channel and polytetrafluoroethylene (PTFE) tubing to the QD channel using a photo-curing glue (Decor Room UV Resin). Galinstan (68.5 %wt Ga, 21.5 %wt In, 10.0 %wt Sn) was inserted into the steel tubes and pressurized with a small weight outside of the chip to ensure that the electrical circuit was not disconnected, as we have previously observed bubbles forming within the galistan channels that break the electrical circuit. This liquid metal channel was used to provide heat to the chip during calibration and operation of the experiment. CdSe/ZnS (Millipore-Sigma, 620 nm peak wavelength) quantum dots that were PEG functionalized were mixed into water before the solution was inserted into the chip via the PTFE tubing. The chip stayed on the glass slide it was printed on, which was held in place by a polylactic acid (PLA) chip holder (Figure 4b). The chip holder has a window above which the chip sits to allow the laser to access the QD channel from below.

A ThorLabs CS235MU black-and-white scientific camera recorded the fluorescent images using a 6 ms exposure time. The schematic in Figure 5a shows the camera was located above the 3D printed chip containing quantum dots, through which the light from the chip would pass through one of six filters (550 nm long-pass, 650 nm ± 20 FWHM, 660 nm ± 5 FWHM, 650 nm ± 5 FWHM, 640 nm ± 5 FWHM, and 620 nm ± 5 FWHM). The filters were switched by a ThorLabs FW103 filter wheel driven by a benchtop stepper motor controller. The quantum dot (QD) channel within the chip was illuminated from below by a 532nm, 2.6 mW laser (OBIS single mode laser from Coherent). A set of galvo mirrors was used to trace the quantum dot channel of the chip with the laser (Figure 5b). The laser scans through a series of 108 positions that trace a serpentine path along the quantum dot channel. Each step takes about 25 *μ*s. During each camera exposure, the laser traces the path once in the forward direction, and then once in reverse to return to its starting point. The entire scan takes just over 5 ms. All the hardware that required synchronization (the camera, filter wheel, and galvo mirrors) were controlled by an Arduino Duo and were hardware triggered (Figure 5c). During post-processing of the images, a 4×4 pixel binning was used to improve the signal-to-noise ratio of each image.

The following procedure was used to record the images so that the temperature of a thermocouple could be used to calibrate the fluorescent emission of the QDs. A thermocouple was placed at the center of the chip, and the chip was placed on the PLA chip holder. The heating channels (containing galinstan) were connected to a benchtop power supply. The chip was then heated using the galinstan channels to a temperature of 30 °C using PID control with the thermocouple providing feedback. Once the thermocouple temperature reading stabilized at 30 °C, the setup was allowed to remain at that set-point temperature for 5 minutes to ensure that the temperature distribution of the chip reached a steady state. Then an image was taken using each of the 6 filters. The temperature setting was then incremented in 10 °C steps to a final temperature of 90 °C, and the process was repeated at each step.

#### 2.2.2 COMSOL Modeling

COMSOL Multiphysics v5.5 was used to simulate the expected temperature distribution within the microfluidic chip. We modified our existing COMSOL model that had been verified previously to represent the current setup of the system [37]. The thermal properties of the chip were from modifications to COMSOL’s material library entry for nylon [37], as our previous independent investigation for the thermal conductivity of PEGDA determined the properties are comparable. As the thermal properties of galinstan are not reported in the literature, it was modeled as liquid gallium, its main constituent. The properties of silica glass, PLA, water, stainless steel, and air were taken from the COMSOL material library. Because the water/QD solutions did not have a high concentration of QDs, the QD channel’s properties were modeled as water. COMSOL simulated Joule heating as a current applied to one of the steel tubes, and the only source of cooling provided was natural convection from the air.

**Fig. 7:**
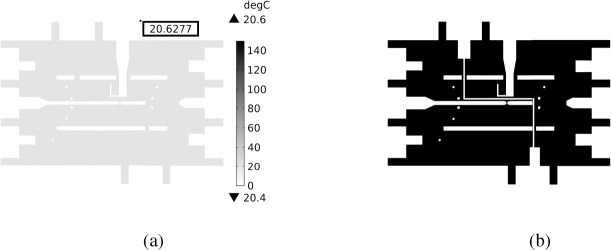
a) Temperature distribution of the plane shown in b) with 20.63 °C representing the temperature of the QD channel closest to the thermcouple location. b) Location of QD channel (the white line that starts from the top and zig-zags to the bottom) where fluorescent emission was during the experiment. The results of the COMSOL model showed that the location of the QD channel closest to the thermocouple location (Figure 6, left) was the same. This is used as a mask for image processing (Section 2.2.3) to limit the fluorescent intensity to only the location of the QDs.

Figure 6 shows the results from COMSOL for low and high heater powers. The green location is highlighting where the thermocouple was located and the temperature at that location, as this would be the place where the experimental temperature would be known and could be compared to the numerical model. Figure 7b shows the temperature distribution (similar to Figure 6, left), where the value of 20.6277 °C is the temperature of the QD channel and is the same as the thermocouple channel.

To match the experimental conditions at each calibration temperature from Section 2.2.1 (nominally 30, 40, 50, 60, 70, 80, and 90 °C), the COMSOL model was used to predict the temperature distribution within the chip when the modeled thermocouple temperature matched the experimentally measured thermocouple temperature.

#### 2.2.3 Production of Dataset #2

**Fig. 8:**
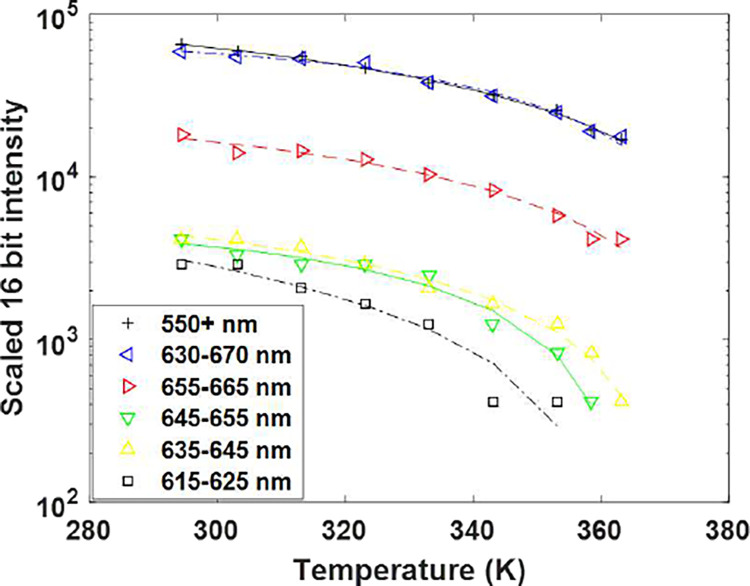
Experimental fit of fluorescent intensities of the QDs to the calibrated temperatures for images taken with each filter.

Dataset #2 consisted of a series of fluorescent images and temperature distribution maps. Because the temperature was known at the thermocouple, the intensity of the image where the QDs were closest to the thermocouple represented the only known location where both fluorescent intensity and temperature were precisely known. These fluorescent intensities/temperature data are shown in Figure 8, with 2^nd^ order polynomial fits superimposed on the data.

**Fig. 9:**
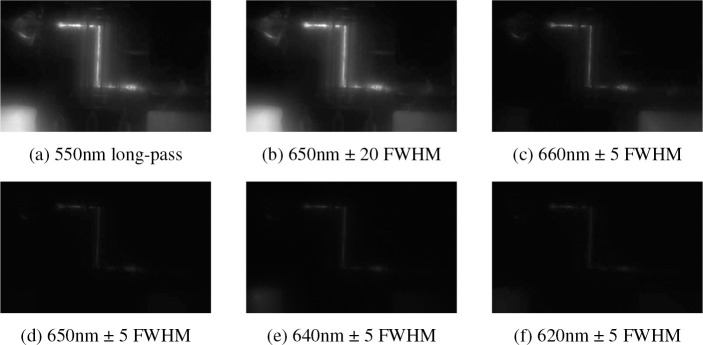
Camera images taken with each filter within the filter wheel.

The mask from Figure 7 was applied to the camera images to isolate the fluorescence of only the QD channel (see Figure 9). The fluorescent data near the thermocouple was recorded and used in creating the models.

The results from the validated COMSOL model were then used to predict the temperature distribution throughout the chip (Figure 6) where there was no fluorescent signal. This provided the ground truth needed for training the neural network. A 2^nd^ order fit was applied to the calibrated fluorescent intensities (scaled to 16-bit values as shown in Figure 8).

## 3 Results

This section will present the results of both LSTM models and a simpler random forest approach to demonstrate the additional accuracy obtained with neural networks.

### 3.1 LSTM Results on Dataset #1

The three networks were trained on the same set of fluorescent images. The FTLSTM had a validation RMSE of ±0.124 K over the entire image. The BFTLSTM had a validation RMSE of ±0.086 K. The MFTLSTM had the best validation RMSE of ±0.0199 K. This is shown in Table 1, where previous neural network architecture results were included to provide a comparison.

**Fig. 10:**
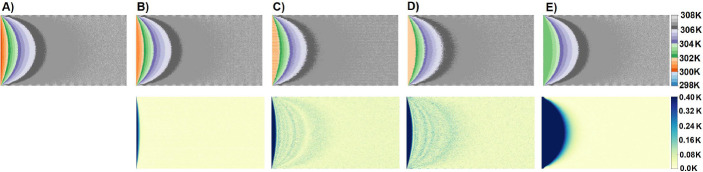
Reconstructing a thermal image. A) Represents the original thermal image which is a simulation of the cross-sectional view of the temperature inside a cylindrical pipe. B) The reconstruction (top) and error (bottom) from MFTLSTM. C) The reconstruction and error from BFTLSTM. D) The reconstruction and error from FTLSTM. E) The reconstruction and error from a multivariate polynomial fit.

Each of the three networks and a multivariate polynomial fit were used to reconstruct a thermal image from fluorescent data (Figure 10). The MFTLSTM had the lowest RMSE of ±0.0509 K (Table 1). The multivariate polynomial fit had the worst RMSE of ±1.21 K (Table 1). That accuracy of nearly ±1 K is consistent with other fluorescent studies that have used polynomial fits [3]. It is interesting to note that as the networks became more complex, their error dropped significantly. This is most like due to the nature of how heat dissipates. The bi-directionality in the BFTLSTM allows the network to consider how heat dissipates in two directions, and the MFTLSTM studies the relationship of the heat dissipating in four directions.

**Fig. 11:**
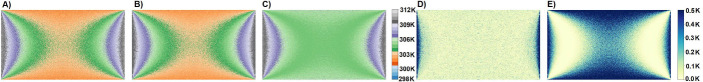
The MFTLSTM was trained on a range of 298–308 K and applied to an image with a temperature range of 298–312 K. The MVPF was fitted to the same training data and then applied to the image with the larger temperature range. A) The target image. B) The MFTLSTM reconstruction. C) The MVPF Reconstruction. D) The MFTLSTM error. E) The MVPF Error.

Unlike the Kullberg [1] study (see Figure 1), our FTLSTM networks were able to accurately reconstruct subtle features at the edges of the image, including the periodic sections on the top and bottom. This is significant because these edges represented some of the area with the highest reconstruction error. The networks were also used to reconstruct a thermal image with a higher temperature range than it was trained on (Figure 11). The MFTLSTM was still reasonably accurate when given temperatures it had not seen before and had an RMSE of ±0.1567 K. The MVPF had the worst results at an RMSE of ±1.307 K. By comparison, our previous work [1] showed significant temperature reconstruction errors when extrapolating or at temperatures where there were fewer than 10^3^ occurrences of that temperature in the total set of images.

Of particular note is that the RMSE of the MFTLSTM is lower than the standard deviation of the ±0.1 K temperature noise imposed on the data in Ref. [1]. One potential reason for this is that during image reconstruction, CNNs have demonstrated the ability to produce de-noised images [40], and our networks have that same capability. When this effect is combined with the long-range spatial relationship mimicing the heat diffusion that is captured in the LSTM structure, we postulate that the network is better able to obtain temperatures close to the ground truth or temperatures that are less affected by measurement noise.

### 3.2 Random Forests Results on Dataset #1

Another non-neural network, machine learning approach was explored for the reconstruction of fluorescent images. Random forests, which are a type of ensemble learning method that consists of multiple decision trees [41], were used. The random forest was trained using the same fluorescent images as the neural networks. When the random forest was given fluorescent images without noise added, the accuracy were comparable to the MFTLSTM’s on noisy images. Yet when noise was added, the accuracy of the random forest was less than the MFTLSTM on the same images. The random forest had an RMSE of ±0.1199 K with the noisy images compared to the ±0.0509 K RMSE the MFTLSTM had. As previously stated, it appears that noise has less affect on the LSTM structure than random forests. While not as accurate as the MFTLSTM, the random forest’s accuracy was near the target ±0.1 K RMSE that is needed, and so it appears to be a possible viable alternative method to the neural networks in certain situations.

### 3.3 LSTM Results on Experiment, Dataset #2

The MFTLSTM was applied to the new experimental data (Dataset #2) from Section 2.2. Only five of the six channels were used to train the network. The 620nm ± 5 FWHM filter was not used as it did not give much additional information to the network. This can be seen in the image taken from that filter in Figure 9. Removing one of the superfluous channels allowed less memory to be used and faster training without sacrificing accuracy. The neural network was trained on temperature data with a range from 290 to 380. This 90 K temperature range the network was trained over is significantly larger than the 10 K [1], 4.4 K [14], and 8 K temperature ranges [42] used in previous papers, and it was able to achieve better accuracy with an RMSE of ±0.0684 K.

When this network was tasked to reconstruct a thermal image of a chip that had a temperature range of 293–294, it had an RMSE of ±0.0241 K (Table 2). As the temperature range on the chip increased, the error increased as well. With a 30 K temperature range (Table 3), the network was still able to get an RMSE less than the goal of ±0.1 K. The largest degree range in a reconstructed image was 50 K. This had an accuracy of ±0.140 K. While this is higher than the goal, it is comparable to the results that FTLSTM, MVPF, and RF had with an image with a temperature range of only 10 K (Table 1).

**Fig. 13:**
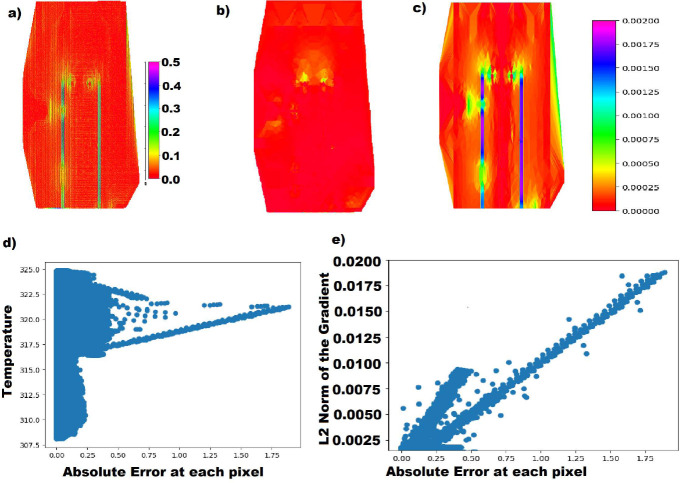
a) The absolute value of the error at every point from a reconstructed temperature image with a temperature range of 308–325 K. b) The absolute value of the Y gradient c) The absolute value of the X gradients, colorbar in K/*μm* d) Temperature at each pixel graphed against the error at that pixel, both in K e) The absolute value of the error in K at each pixel graphed against the L2 norm of the gradient vectors.

There are several possible reasons for the increased error. First, there is less variance in the fluorescent data as the temperature rises, as can be seen in Figure 8. Another reason for the error increase could be due to the larger temperature gradient around the heat sources on the chip. From Table 2, it can be seen that the error is highest in a straight vertical line where the heat sources are located. When comparing the error images to an image of the temperature gradient of the chips, there is a clear visual correlation between the error and temperature gradient (Figure 13). This gradient gets larger as the temperature range increases. For instance, the chip with a temp range of 293K to 294 K had an average L2 norm of the gradient vector of 2.91 10^−6^ K/*μm*, the chip with a temp range of 308 K to 325 K had a mean L2 norm gradient vector of 2.38 10^−4^ K/*μm*, and the chip with a temp range of 327K to 372 K had a mean L2 norm gradient vector of 6.43 10^−4^ K/*μm*. The max L2 norms of the gradients of the three images were 2.38 10^−4^ K/*μm*, 1.88 10^−2^K/*μm*, and 4.91 10^−2^K/*μm*, respectively. The 50 K range image has an average L2 norm that is 221 times larger than the 1 K range. This larger gradient seems to cause a larger error as there is a significantly larger difference in temperatures from pixel to pixel. Lastly, The optical properties of the resin by the heat sources may be different than the rest of the channel, causing a blur effect and creating artifacts.

## 4 Conclusions

Using literature data on fluorescent spectra as a function of temperature, a series of temperature maps were created and partitioned. Using a fluorescent thermometry long-term/short-term memory (FTLSTM) neural network to analyze the images, an RMSE of ±0.124 K was obtained, similar to the ±0.1 K Gaussian noise added to the original temperature distributions of the synthetic data (Dataset #1). This low value is significant because the network improved the temperature accuracy despite the additional noise included when creating the fluorescent images. This accuracy was further improved to ±0.086 K with a Bi-Direction FTLSTM and to ±0.0199 K with a Multi-Directional FTLSTM.

Fluorescent images of a microfluidic chip were taken experimentally, and the ground truth temperature distribution of the chip was determined by a combination of experimental temperature measurements and a validated COMSOL model. The Multi-Directional LSTM was trained on a temperature range of 290 – 380 K and had an accuracy of ±0.0684 K RMSE with this new dataset (Dataset #2).

When this network was tasked with reconstructing the temperature images of these chips, it had an accuracy better than ±0.1 K RSME when the temperature range of the image was 30 K or less. The smaller temperature range presented on the chip led to increased accuracy.

We predict that the improvement in temperature accuracy is based on the FTLSTM’s ability to incorporate the spatial distribution of data, use the predicted relationship in one portion of the image, and apply it to other portions of the image.

## Figures and Tables

**Fig. 12:**
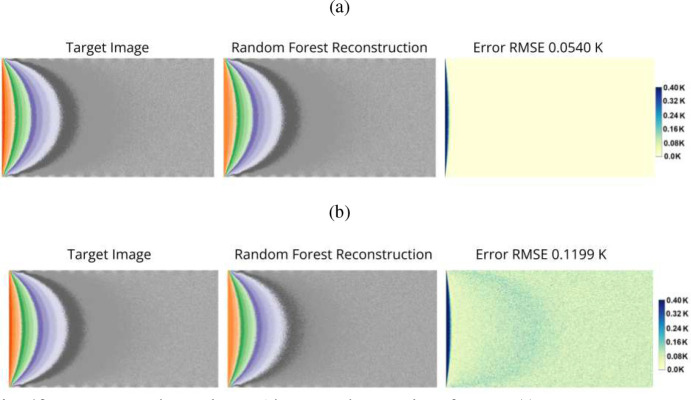
Reconstructing a thermal image using random forests. A) Represents reconstructing a thermal image with no noise added using random forests (RF No Noise). B) The reconstruction of a thermal image with noise added using random forests (RF Noise in Table 1.

**Table 1:** Results of multiple NN, random forest (RF), and multivariate polynomial fit (MVPF) on the same Dataset #1 images. Results from a simply connected feed-forward NN (SCFF), modified U-net (MU-net), and 5-channel modified convolutional neural network (5MCNN) are from previous work [1].

Network	Validation RMSE	Figure 10 and 12	Figure 11
SCFF	±33.9 K [1]	-	-
MU-net	±1.09 K [1]	-	-
5MCNN	±0.23 K [1]	-	-
MVPF	-	±1.2089 K	±1.307 K
RF No Noise	-	±0.054 K	-
RF Noise	-	±0.1199 K	-
FTLSTM	±0.124 K	±0.2021 K	±0.4114 K
BFTLSTM	±0.086 K	±0.1569 K	±0.3339 K
MFTLSTM	±0.0199 K	±0.0509 K	±0.1567 K

**Table 2:** Reconstructing the temperature map of the microfluidic chip when the chip was at different temperature ranges. All color bars are in units of K.

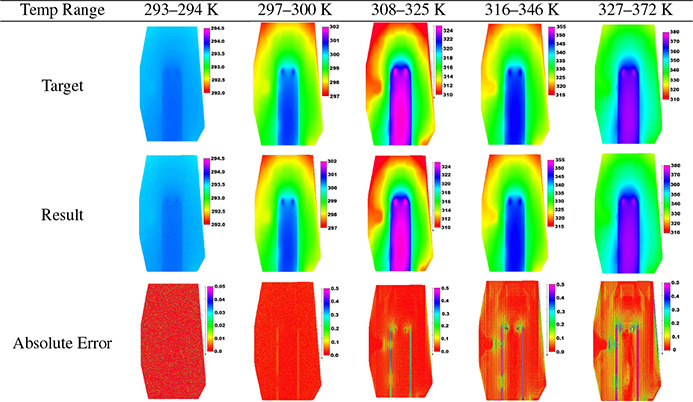

**Table 3:** The RMSE from recreating the temperature map on the chip data at different temperature ranges

Temp Range	293 to 294 K	297 to 300 K	308 to 325 K	316 to 346 K	327 to 372 K
RMSE	±0.0241 K	±0.030 K	±0.0574 K	±0.0945 K	±0.140 K
